# Birds and plastic pollution: recent advances

**DOI:** 10.1186/s40657-021-00293-2

**Published:** 2021-11-02

**Authors:** Limin Wang, Ghulam Nabi, Liyun Yin, Yanqin Wang, Shuxin Li, Zhuang Hao, Dongming Li

**Affiliations:** Ministry of Education Key Laboratory of Molecular and Cellular Biology, Key Laboratory of Animal Physiology, Biochemistry and Molecular Biology of Hebei Province, College of Life Sciences, Hebei Normal University, Shijiazhuang, 050024 China

**Keywords:** Birds, Microplastics pollution, Plastics pollution, Toxicological effects

## Abstract

Plastic waste and debris have caused substantial environmental pollution globally in the past decades, and they have been accumulated in hundreds of terrestrial and aquatic avian species. Birds are susceptible and vulnerable to external environments; therefore, they could be used to estimate the negative effects of environmental pollution. In this review, we summarize the effects of macroplastics, microplastics, and plastic-derived additives and plastic-absorbed chemicals on birds. First, macroplastics and microplastics accumulate in different tissues of various aquatic and terrestrial birds, suggesting that birds could suffer from the macroplastics and microplastics-associated contaminants in the aquatic and terrestrial environments. Second, the detrimental effects of macroplastics and microplastics, and their derived additives and absorbed chemicals on the individual survival, growth and development, reproductive output, and physiology, are summarized in different birds, as well as the known toxicological mechanisms of plastics in laboratory model mammals. Finally, we identify that human commensal birds, long-life-span birds, and model bird species could be utilized to different research objectives to evaluate plastic pollution burden and toxicological effects of chronic plastic exposure.

## Background

**Fig. 1:**
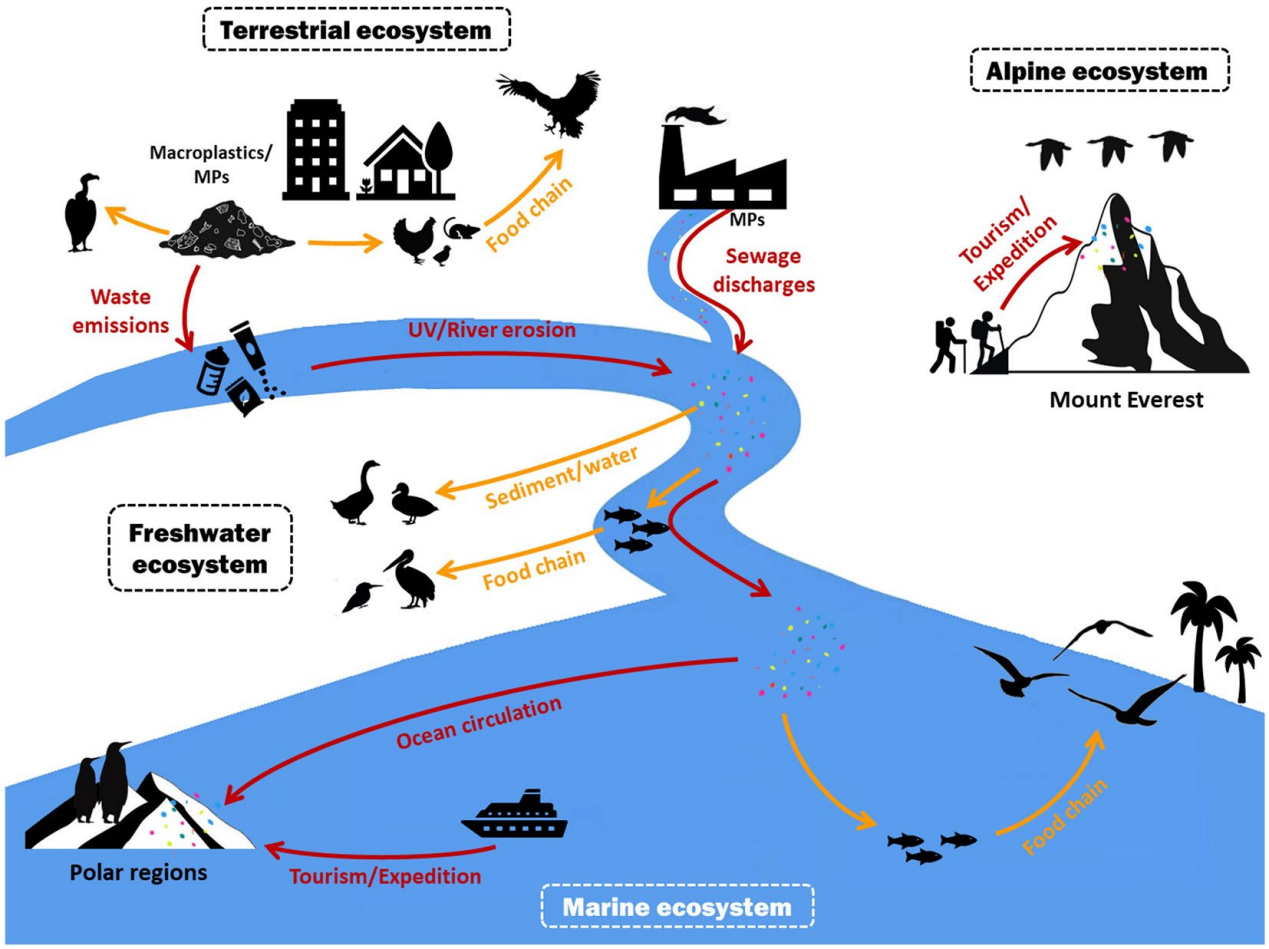
The cycling process of macroplastics and microplastics in different ecosystems (red arrow) and potential uptake ways by birds from different ecological groups (orange arrow)

Along with global industrialization and modernization, the production and consumption of plastic items have increased substantially since the early 1950s (Geyer et al. 2017; MacLeod et al. 2021). Approximately, 8.3 billion metric tons of virgin plastic were produced up to 2017, and 12 billion tons of plastic wastes are expected to be found in the natural environment by 2050 (Geyer et al. 2017). Most plastic products (macroplastics, diameter > 5 mm) are not biodegradable and break down into small plastic particles that can be easily spread to various environments by the action of wind and waves owing to their small size, lightweight, high durability, and extended stability (Susanti et al. 2020). In recent years, plastic particles with diameter ≤ 5 mm (microplastics, MPs) and ≤ 1 μm (nanoplastics, NPs) have been increasingly observed in various compositions, shapes, morphologies, and textures in atmospheric, terrestrial, and marine environments, and they can enter the food chain either by inhalation or by ingestion (Susanti et al. 2020; Fig. 1). MPs have also been discovered in remote areas such as polar regions (Bessa et al. 2019), Mount Everest (Napper et al. 2020), and the Mariana Trench (Jamieson et al. 2019). MPs can act as vectors for pathogens and chemical pollutants because of their environmental persistence and potential ecotoxicity, which pose significant health and ecological concerns (Amelineau et al. 2016; Nabi et al. 2019). Furthermore, they are bioavailable for ingestion by a variety of wild organisms (Cole et al. 2013; Bessa et al. 2018; Nelms et al. 2019) and can enter food chains through trophic transfer, causing severe threats to biodiversity and ecosystems (Karami et al. 2016; Dawson et al. 2018; Zhu et al. 2018). Therefore, the accumulation of plastic waste and debris in the environment has continuously increased, resulting in substantial environmental pollution (Rochman et al. 2013; Wilcox et al. 2015; Zhu et al. 2019).

Birds have the largest number of species (more than 10,000 living species) among the tetrapod classes (Ducatez and Lefebvre 2014). They are endotherms organisms that are widely distributed in various habitats worldwide, from the equator to polar areas, and from oceans and freshwater to high plateaus, and they exhibit flight-related morphological and physiological traits that enable them to occupy different habitats and become important members of many ecosystems (Orme et al. 2006) (Fig. 1). Compared with non-flying animals, birds have a higher metabolic rate (McNab 2009), better antioxidant capacity (Costantini 2008), prolonged lifespan (Munshi-South and Wilkinson 2010) and short but efficient digestive tract (Caviedes-Vidal et al. 2007). They are believed to be highly sensitive and vulnerable to external conditions, and therefore, could be used to monitor environmental changes and assess the negative effects of environmental pollution (Carral-Murrieta et al. 2020; Li et al. 2021; Nabi et al. 2021). Given that birds in particular mistake plastic for prey, macroplastics or MPs have been found in the gastrointestinal tracts, feces, and even in feathers and other tissues or organs of several hundred avian species from freshwater, terrestrial, and marine ecosystems (Carey 2011; Gall and Thompson 2015; Wilcox et al. 2015; Zhao et al. 2016). Here, we review the occurrence of plastics and MPs in aquatic and terrestrial birds (Fig. 1); summarize the effects of plastics, MPs, plastics-derived additives, and plastic-absorbed chemicals; and suggest directions for further research in the field of plastic pollution in birds.

### Macroplastics and microplastics in aquatic and terrestrial birds

Plastic debris is ubiquitous in oceans, and its potential impacts on a wide range of marine organisms have raised serious concerns (Andrady 2011; Jambeck et al. 2015; Yin et al. 2018, 2019). Globally, the proportion of MPs to the total weight of plastic accumulated in the environment by 2060 is estimated at 13.2% (Andrady 2011). Macroplastics and MPs in the oceans are similar in size and appearance to tiny marine organisms (e.g., zooplankton), and they can be wrongly regarded as prey by marine animals such as fish and shellfish (Waring et al. 2018). These marine animals are the primary food resource of many seabirds, so that the seabirds are particularly susceptible to plastic exposure because of their high rates of ingestion of contaminated prey (Barbieri et al. 2010). It is estimated that up to 78% of identified species of seabirds have deposited MPs in their digestive tracts since the 1960s (Wilcox et al. 2015; Basto et al. 2019), and more than 99% of over 300 seabird species are expected to have ingested plastic debris by 2050 (Wilcox et al. 2015). The positive correlation between MPs in feathers and fecal samples in geese and ducks (Reynolds and Ryan 2018) suggests that MPs can accumulate in different tissues of their bodies. Seabirds spread particulate plastics at colonies through regurgitation (Lindborg et al. 2012; Hammer et al. 2016) and guano deposition, thereby increasing the concentration of chemical contaminants near their colonies (Blais et al. 2005). Therefore, seabirds function as vectors for marine-derived MPs and plastic-associated contaminants in the aquatic and terrestrial environments.

Terrestrial birds are an essential component of land ecosystems, with various ecological functions in the food web (Carlin et al. 2020). Zhao et al. (2016) reported that MPs were discovered in the gastrointestinal tracts of 16 out of 17 terrestrial bird species. Unlike many studies on aquatic birds, there are few studies on terrestrial birds, except for plastic ingestion by several top bird predators (Carlin et al. 2020; Ballejo et al. 2021). The occurrence of macroplastics and MPs has been reported in some raptors, because raptors are top predators, and has relatively large foraging areas, and a longer lifespan (Houston et al. 2007; Carlin et al. 2020; Ballejo et al. 2021). For instance, the California Condor (*Gymnogyps californianus*), a critically endangered species, has been reported to ingest plastic from rubbish dumps (Houston et al. 2007), which is considered one of the most important causes of death in nestlings (Rideout et al. 2012). In addition, another study showed that MPs were significantly more abundant in the digestive tract tissue of Red-shouldered Hawk (*Buteo lineatus*), that consumes small mammals, snakes, and amphibians, than in fish feeding Osprey (*Pandion haliaetus*) (Carlin et al. 2020). Vultures are obligate scavengers, and many of them use rubbish dumps as food resources worldwide, including the Andean Condor (*Vultur gryphus*), Black Vulture (*Coragyps atratus*), and Turkey Vulture (*Cathartes aura*) (Houston et al. 2007; Plaza et al. 2018; Carlin et al. 2020; Ballejo et al. 2021). This feeding habit increases their exposure risks to MPs consumption through organic waste and synthetic materials, which can cause intestinal obstructions, nutritional problems, infections, and metabolic alterations (Plaza et al. 2018; Tauler-Ametlller et al. 2019). Although small-sized terrestrial birds (e.g., passerines) are highly diversified and widely distributed relative to raptors (Yu et al. 2014), little is known about the relationship between the occurrence of macroplastics and MPs in small-sized terrestrial birds.

### Effects of macroplastics and microplastics on birds

**Fig. 2:**
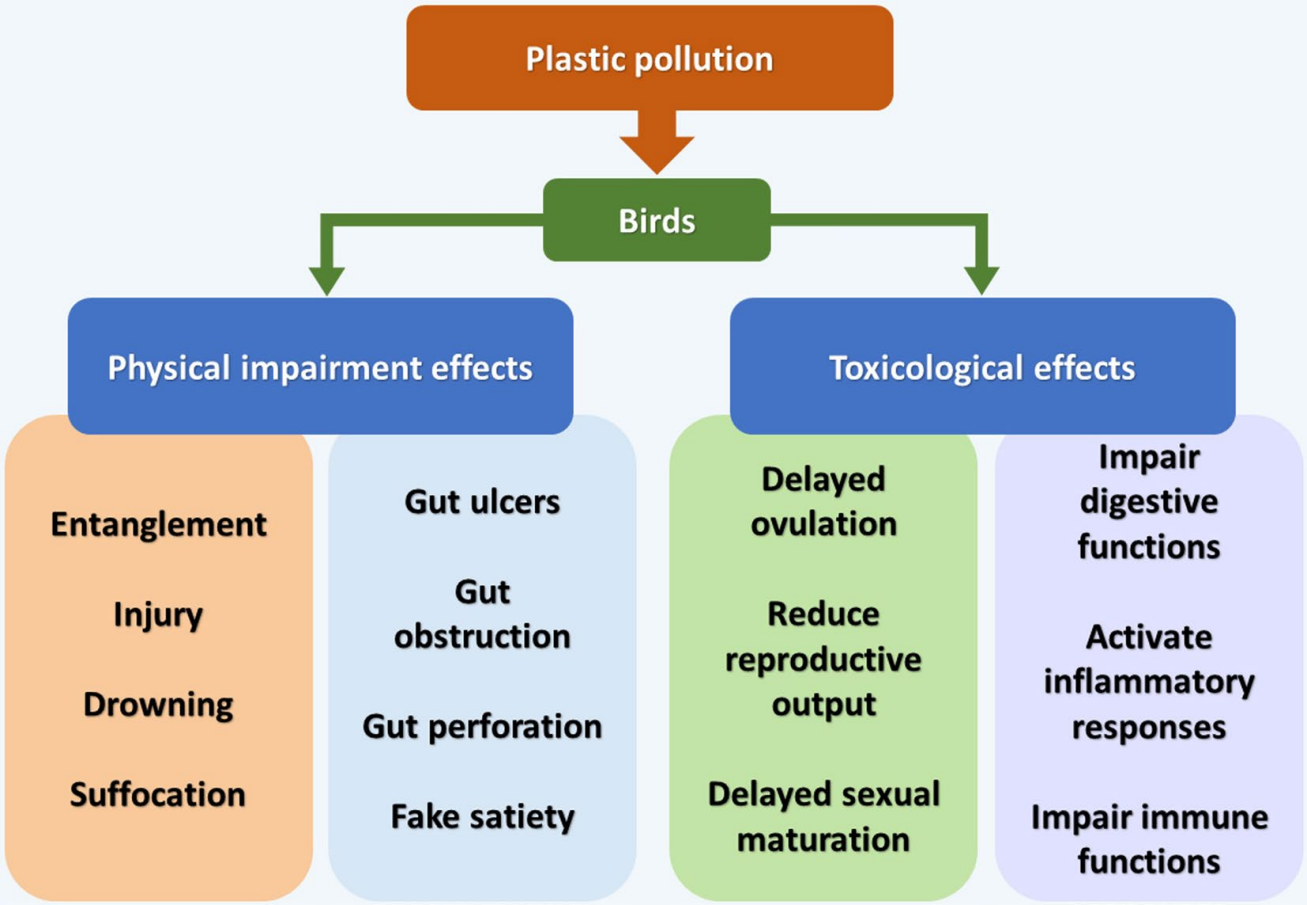
The physical impairment and toxicological effects of environmental plastic pollution on birds

Various negative consequences are resulting from interactions between wildlife and plastic debris. The most obvious and immediate consequences include entanglement (Derraik 2002; Ryan 2018; Lavers et al. 2020), nutritional deprivation (Lavers et al. 2014), and damage or obstruction of the gut (Pierce et al. 2004). Particularly, more and more birds are severely affected by entanglement owing to the increasing presence of plastic litter (Gregory 2009; Roman et al. 2019), e.g., the large number of face masks carelessly discarded during the COVID-19 pandemic (Patrício Silva et al. 2021). Entanglement can lead to injuries, drowning, and even suffocation, which can reduce predation efficiency and increase the probability of being preyed upon (Derraik 2002; Gall and Thompson 2015). Furthermore, large plastic fragments and tiny plastic particles are also frequently ingested by birds (Derraik 2002; Ryan 2018; Lavers et al. 2020). For example, microplastic fibers, beads, and macroplastics have been found embedded in the intestinal wall of Red-shouldered Hawk and Osprey, which suggests that these materials can remain in the intestines longer than other indigestible items that pass through (Carlin et al. 2020). Several pioneering studies have reported that the deposited and aggregated MPs or larger plastic debris can cause bleeding, blockage of the digestive tract, ulcers, or perforations of the gut, which can produce a deceptive feeling of satiation (Derraik 2002; Pierce et al. 2004), lead to starvation (Derraik 2002; Pierce et al. 2004), or cause direct mortality (Derraik 2002; Roman et al. 2019). For example, the volume of plastic ingested (plastic burden) by the Northern Gannet (*Morus bassanus*) and the Great Shearwater (*Puffinus gravis*) can be associated with damage or obstruction of the gut, reduced body weight, slower growth rate, and increased mortality (Pierce et al. 2004). Similarly, a decreased growth rate induced by plastic ingestion was observed in the chicks of Flesh-footed Shearwater (*Puffinus carneipes*) (Lavers et al. 2014) and Japanese Quail (*Coturnix japonica*) (Roman et al. 2019), which likely resulted from reduced stomach capacity rather than toxicological effects (Fig. 2).

Some studies have found that ingestion of MPs has reproductive toxicity to birds (Fossi et al. 2018; Roman et al. 2019). For example, chicks of Japanese Quail with observed plastic ingestion exhibited a minor delay in sexual maturity, and a higher incidence of epididymal intra-epithelial cysts in males, although there were no effects on reproductive success (Roman et al. 2019). Similarly, the ingestion of MPs can also reduce the reproductive output of Flesh-footed Shearwater (Fossi et al. 2018). Carey (2011) observed that the plastics or microplastics ingested by adult Short-tailed Shearwater (*Ardenna tenuirostris*) could be passed to their chicks. Furthermore, ingestion of MPs by birds can activate inflammatory responses, and lead to reducing food intake, delayed ovulation, and increased mortality (Wright et al. 2013; Carbery et al. 2018; Fossi et al. 2018) (Fig. 2). In this context, it is important to determine the potential MPs concentration that is detrimental or sublethal to body condition, development, growth, reproduction, and other physiological functions in birds (Puskic et al. 2019).

### Effects of plastics-derived additives and plastics-adsorbed chemicals on birds

Plastic debris contains a wide range of additives and toxic chemicals sorbed from the environment (Hirai et al. 2011), which can have various adverse effects on wildlife organisms (Chen et al. 2019; Tanaka et al. 2020). The European Chemicals Agency has listed approximately 400 plastic additives, including organotins, triclosan, phthalates, brominated flame retardants, bisphenols, and diethyl hexyl phthalate (DEHP) (Du et al. 2017; Hermabessiere et al. 2017; Zhang et al. 2018). The accumulation of plastic additives has been reported in several seabirds, including the Streaked Shearwater (*Calonectris leucomelas*) (Teuten et al. 2009), Short-tailed Shearwaters (Yamashita et al. 2011), and Flesh-footed Shearwaters (Lavers et al. 2014), suggesting that plastics are a direct carrier of chemicals to seabirds. Among these chemicals, many studies confirm that DEHP can cause weight gain in European Starling (*Sturnus vulgaris*) (O’Shea and Stafford, 1980) and is potentially toxic to the kidneys (Li et al. 2018), liver (Zhang et al. 2018), and cerebellum (Du et al. 2017) in Japanese Quail.

In addition, owing to their hydrophobic nature and relatively large surface area, MPs can adsorb numerous environmental contaminants, such as POPs, heavy metals, polycyclic aromatic hydrocarbons (PAHs), polychlorinated biphenyls (PCBs), antibiotics, and endocrine-disrupting compounds (EDCs) (Rathi et al. 2019; Reddy et al. 2019). Previous studies have shown that ingestion of toxic substances adsorbed on MPs can induce malnutrition, endocrine disruption, and issues in the reproductive biology of Japanese Quail (Roman et al. 2019) and several species of seabirds, including Kelp Gull (*Larus dominicanus*) (Barbieri 2010), Short-tailed Shearwater (Tanaka et al. 2013), White-chinned Petrel (*Procellaria aequinoctialis*), Slender-billed Prion (*Pachyptila belcheri*), Great Shearwater, Black-browed Albatross (*Thalassarche melanophrys*), and Southern Giant Petrel (*Macronectes giganteus*) (Susanti et al. 2020). Chronic exposure to EDCs can have several negative effects on the developmental and reproductive biology of Japanese Quail (Ottinger et al. 2008), Tree Swallow (*Tachycineta bicolor*) (McCarty and Second 2000), American Kestrel (*Falco sparverius*) (Fisher et al. 2001), Great Blue Heron (*Ardea herodias*) (Sanderson et al. 1994) and White Ibis (*Eudocimus albus*) (Jayasena et al. 2011), and it also can impair immune and thyroid functions in Japanese Quails (Ottinger et al. 2008). Furthermore, EDCs cause poor reproductive output because of embryonic death, chick deformities, eggshell thinning, and even death in Japanese Quails (Ottinger et al. 2005). Previous studies have shown that traditional pollutants, such as heavy metals and organic pollutants (POPs) are detrimental to the health of birds. For example, heavy metals have adverse effects on the testicular function and sperm quality of Eurasian Tree Sparrows (*Passer montanus*) (Yang et al. 2020) and White Ibises (Frederick and Jayasena 2011), and POPs exert numerous negative effects on endocrine, immune and neural system in White-tailed Eagle (*Haliaeetus albicilla*) (Sletten et al. 2016) and reproduction, and development, and growth in other bird species (Hao et al. 2021). However, it is quite challenging to find pertinent data for each toxicant because of the large number of plastic-associated toxicants identified in wild avian species.

### Known toxicological and physiological effects of macroplastics and microplastics in other animals

Plastic debris and MPs have also been found in the digestive tracts of a variety of animal groups from various environments. First, plastics can cause entanglement or lead to starvation or intestinal blockages upon ingestion (Gregory 2009; Provencher et al. 2017). Second, MPs can be deposited in the mucus layer secreted by the cells of the gut wall, and then transported to other organs or tissues via circulation (Lu et al. 2018; Jin et al. 2019). In addition to the physical impairment and histological variations in the intestines, the perils of MP ingestion include growth impediment and disorders of metabolism and behavior (Lu et al. 2018; Jin et al. 2019). MPs also impair filter feeders (mussels and clams) and induce DNA damage, oxidative injury, and antioxidative responses (clams) (Cedervall et al. 2012; Ribeira et al. 2017). Furthermore, endocrine disruption and neurotransmission dysfunction of marine species caused by MPs have been reported, in addition to genotoxicity (Rochman et al. 2014; Avio et al. 2015). Polystyrene MPs can adversely affect granulocytes and ovarian function in female rats through distinct signaling pathways (Hou et al. 2021).

Compared with plastic debris and MPs, NPs have a higher potential to negatively affect organisms because they can penetrate and accumulate in organs or tissues through systemic circulation (Kashiwada 2006; von Moos et al. 2012) and even pass biological barriers (Mattsson et al. 2016; Borisova 2018). NPs can interact with proteins, lipids, and carbohydrates, which affect transmembrane transport (Revel et al. 2018) and metabolism (Cedervall et al. 2012; Mattsson et al. 2015), and can lead to reproductive dysfunction and behavioral abnormalities in aquatic (Chae and An 2017; Mattsson et al. 2017; Prüst et al. 2020) and terrestrial (Amereh et al. 2020; Prüst et al. 2020) animals. Furthermore, NPs have induced adverse effects on the reproductive functions of laboratory mammals (Amereh et al. 2020; An et al. 2021; Jin et al. 2021), such as alterations in sperm morphology and viability, and lower serum testosterone, luteinizing hormone (LH), and follicle-stimulating hormone (FSH) in mice and rats (Amereh et al. 2020). Polystyrene NPs can cause depression and behavioral and cognitive disorders in mice (da Costa Araújo and Malafaia 2021; Estrela et al. 2021). Despite the limited information on the toxicological effects of NPs on non-laboratory model animals, the above-mentioned effects of widely distributed NPs can be inferred to occur in free-living animals.

## Future directions

The increasing demand for plastic products coupled with inadequate waste management and policy contributes to the ongoing and rapidly expanding environmental pollution of plastics (Rochman et al. 2013; Borrelle et al. 2017). MPs are hazardous not only to birds but also to other animals, including humans. In recent years, an increasing number of studies have identified the occurrence of plastics and plastics-associated toxicants in various animals associated with a significant increase in plastic pollution. Although an increasing number of studies have focused on the phenomenon of plastic deposition and toxicological effects in birds, the mechanisms throughout which MPs enter tissues and their potential health risks have not been fully clarified. Although MPs do not exhibit apparent toxicity, they can absorb toxic chemicals, which further challenges our understanding of the overall impacts of MPs. Further investigations are needed to determine whether the endocrine and toxicological effects of MPs-related contamination (e.g., plastics-derived additives and plastics-adsorbed chemicals) occur in wild birds with sufficient severity to be detrimental to fitness, and whether birds suffer ongoing disadvantages upon chronic low-level toxicity.

As birds have a great number of specific groups, different groups can be used to assess the plastic pollution burden, long-term effects of MPs exposure in various environments, and toxicological effects in the laboratory. For instance, human commensal species, such as the Eurasian Tree Sparrow (Sun et al. 2016; Li et al. 2019; Yang et al. 2019; Ding et al. 2021), House Sparrow (*P. domesticus*) (Hanson et al. 2020) and House Wren (*Troglodytes aedon*) (Juárez et al. 2020) utilize human resources in rural and urban areas and have a remarkably broad distribution range. These species could be used as bioindicators to evaluate the plastic pollution burden in different environments because they have been well studied in the past two decades. In addition, as long-lifespan species (e.g., albatrosses, shearwaters, and vultures) can breed over many decades (Moore 2008), they could be used to evaluate the potential toxicological effects of chronic plastic exposure on both individual survival and reproductive output (Kramar et al. 2019; Marín-Gómez et al. 2020; Sánchez et al. 2020). Furthermore, these species could be used to evaluate the effects of food contaminated with plastic debris and the intergenerational transfer of MPs through allofeeding of offsprings (Sánchez et al. 2020), as observed in the Cory’s Shearwater (*Calonectris diomedea*) fledglings (Rodríguez et al. 2012), Providence Petrel (*Pterodroma solandri*) (Bester et al. 2010), Black-footed Albatross (*Phoebastria nigripes*) (Rapp et al. 2017), Laysan Albatross (*P. immutabilis*) (Young et al. 2009), Short-tailed Shearwater (Carey 2011), Wedge-tailed Shearwater (*A. pacifica*) (Verlis et al. 2013), Flesh-footed Shearwater (Lavers et al. 2014), and other petrels (Rapp et al. 2017). Finally, model bird species (chicken and Japanese Quail) could be used to clarify the potential regulatory mechanisms associated with physiology, behavior, and neuroendocrinology upon exposure to different sizes of MPs.

NPs can cause more potent threats than MPs to mammals because they are small enough to accumulate in different tissues through systemic circulation (Estrela et al. 2021). In birds, one can predict that NPs might cause behavioral, physiological, and neuroendocrinological changes, although there has been no identified evidence, and further investigations are necessary. Furthermore, as birds build nests with many natural and human-related materials, the potential threat of plastic debris, MPs, or NPs as nest materials to embryonic and chick development needs to be further examined. Birds are unique and differ from other animal groups because of their behavior, physiology, and lifestyle. Further research should focus on the underlying toxicological mechanisms of MPs and NPs in the laboratory or free-living birds and the identification of consistent and inconsistent response mechanisms to plastics-related pollution (i.e., macroplastics, MPs, NPs, plastics-derived additives, and plastics-adsorbed chemicals) in birds and other animal groups.
